# Comparison of Quality of Life Following Single- Event Multilevel Surgery (SEMLS) Using Bandaging and Casting Immobilization Methods in Cerebral Palsy Children

**DOI:** 10.22037/ijcn.v15i2.17361

**Published:** 2021

**Authors:** Esmaeil SADEGHI, Ali Asghar JAMEBOZORGI, Mohamad QOREISHY, Melika KANGARANI FARAHANI

**Affiliations:** 1 Occupational Therapy, Iran University of Medical Sciences, Tehran, Iran; 2 Physiotherapy Research Center, School of Rehabilitation , Shahid Beheshti University of Medical Sciences, Tehran, Iran; 3 Orthopedic Surgen of Hip & Pelvis Fellowship, Shahid Beheshti University of Medical Sciences, Tehran, Iran; 4 Rehabilitation Sciences, British Columbia University, Vancouver, Canada

**Keywords:** Cerebral palsy, Bandage, Casting, Quality of life, Surgery

## Abstract

**Objective:**

Cerebral palsy (CP) is a non-progressive Neurodevelopmental disorder mainly treated using Single-event multilevel surgery (SEMLS). SEMLS contains using a casting method to immobilize the operated limb. However, in the present study, in addition to casting, the bandaging method was also applied. Bandaging is a newer method compared to casting. No study has used bandage for post-surgery immobilization. According to the best knowledge of the authors, no study has compared the outcome of bandage and cast for postoperative immobilization regarding the rehabilitation and quality of life (QoL) in the first and third months following the surgery, within the recovery period, which is associated with consequences like caring, hygiene, transferring, and mobility that affect the spirit and function of children. As a result, we decided to investigate the effect of these methods on the QoL of children the following surgery to treat CP.

**Materials & Methods:**

Following an analytical cross-sectional design, 100 children (aged 4-12 years) were randomly divided into hemiplegic and diplegic CP. The Cerebral Palsy QoL questionnaire (CP QOL-Child) was filled by parents of the participants. Based on the type of administered immobilizer, 80 children were randomly divided into two groups (40 subjects in each group). All subjects were evaluated using a similar questionnaire in the first and third months after surgery. The non-parametric Mann-Whitney test and ANOVA test were used to compare the study groups.

**Results:**

The mean ratio of QoL changes, based on the CP QoL-Child questionnaire, was significantly increased in the bandage group during the first month after surgery. However, for the cast group, this parameter was significantly decreased (P<0.001). In the third month after surgery, the mean ratio of QoL changes was significantly increased in both groups, but the difference in the mean ratio of QoL changes between the two methods wasn’t significant (P=0.64).

**Conclusion:**

In the first month after surgery, the bandaging method was more effective than the casting method, but in the third month, the outcomes were similar for both groups.

## Introduction

Cerebral palsy (CP) is a non-progressive neurodevelopmental disorder caused by damage to motor areas of the developmental brain during fetal development, birth, or in the first few years of life that result in limitations in various activities particularly voluntary movements (1-4). The motor impairments of CP are often accompanied by other disturbances such as sensation, cognition, communication, perception, behavior, seizures, and secondary musculoskeletal problems (5). Classification of the CP is based on the anatomic distribution into monoplegia, hemiplegia, diplegia, and quadriplegia. In cases with diplegia, lower extremities are more affected than the upper extremities, while hemiplegia is associated with the involvement of one side of the body (6). 

Little (1958) reported a prevalence of 2.5 per 1,000 live births for CP, which decreased to 1.5 fifteen years later. In a survey study by Winter (2002), which is conducted in five American countries, a prevalence of 2 per 1000 live birth is reported for CP. Surman et al. (2003) (In England) and Kate Himmelmann (2006) (in Sweden) reported a prevalence of 1.7 and 1/92 per 1000 births, respectively (7). In Iran, a prevalence of 2 per 1000 children is reported (8). 

Currently available therapeutic options for CP include medical interventions, rehabilitation therapies, and complex orthopedic interventions. Also, orthopedic surgeries are often used along with other treatments in order to prevent or correct secondary musculoskeletal problems in children with CP (9). The Single-Event Multilevel Surgery (SEMLS) approach is an effective strategy that comprises lengthening, transfer, and correction of deformities in one surgical session (10). SEMLS surgery is associated with reduced length of hospital stay, improved gait pattern, enhanced range of motion of joint, changes in the X-ray results, increased energy conservation as well as improved functional outcome (11-17). 

Children with CP who had SEMLS surgery not only will experience better motor function but also will have improved quality of life (QoL) (18). In this type of surgery, the casting method is generally used for postoperative immobilization, but in the current study, the bandaging method was applied in addition to the casting (19). Lubicky et al. (2003), in a study intended to compare the casting method with a non-casting method, reported that the former resulted in more skin sores, wound infection, re-operation, and re-hospitalization rate than the latter. However, the difference was not statistically significant. It worth noting that they didn’t declare another method of immobilization. Apart from its ease of administration, casting promotes the healing process of osteotomies, prevents instrumentation failure, and protects the operated leg from injury. Meanwhile, it has some particular consequences like poor hygiene, bone pain following cast removal, scarring, and stiffness of joints, mainly as a result of immobilization (20). 

In this study, the SEMLS surgery was performed in diplegic and hemiplegic CP children, preferably not older than 4 years, and the bandage was applied as an immobilizer. The SEMLS surgery consisted of Achilles Tendon Lengthening (ATL), by means of open sliding technique through 1 to 2 inches incision, muscles lengthening (lengthening of Semitendinosus, Semimembranosus and Gracilis muscles) in order to correct knee flexion deformities, and Longus muscle releasing in the hip adductor, only by making one incision until affected hip reached to 30-40 degree passive abduction. After the SEMLS surgery, the surgical area was covered with a bandage. For bandaging, sterile gauze and webril pad were placed over the surgical area. Afterward, the bandage was applied from distal to proximal. Bandaging was performed to provide the appropriate support of the area, and occupational therapy began the day after surgery. There has been no interest in long-term immobilization after surgery, mainly because of its complications (e.g., muscle weakness, joint contracture, and delayed recovery and rehabilitation process, as well as returning to daily activity without limitation) (21).

Our literature review revealed no study on comparison of bandage and cast with respect to the rehabilitation, and it is not clear which of these immobilizers is more appropriate in terms of improving the QoL. Hence, the current study aimed to compare the cast and bandage methods for postoperative immobilization in children with spastic diplegia CP in terms of prevention of musculoskeletal problems caused by immobility, in order to reach the following objectives: to decrease the required time for a rehabilitation program, to improve the function, and to facilitate a faster return to daily activities. The minimum time that is considered in previous studies to evaluate the QoL of children has been nine months. However, two time points of one and three months after the surgery were considered. In fact, the study was performed in the recovery period, which is accompanied by some common problems like caring, hygiene, transferring, and mobility, which in turn affect both the spirit and daily function of children. 

## Materials & Methods

A total of 80 children (aged 7 to 12 years) with spastic diplegic and hemiplegic CP, with the ability of independent movement, without a history of surgery, were recruited. Only those who had at least one of these criteria were recruited: fixed contractures (angle of hip abduction<30 degrees without hip dislocation risk and angle of fixed flexion hip and knee joints > 10-15 degree.), hindfoot varus, hindfoot valgus, progressive equinus hindfoot deformity combined with hindfoot, and talus fracture. 

Participants were selected from a population of individuals with CP referred to the orthopedic department of Akhtar hospital. All subjects were evaluated by a team of surgical specialties, and the goniometric measurements were also performed. In the present study, the objectives of the study were explained to the parents of eligible children, and parental informed consent was obtained for all cases. In addition, medical ethics principles were followed, and no financial burden was imposed on participants due to participating in the study. Data were collected using the Cerebral Palsy QoL Questionnaire for Children (CP QOL-Child). This questionnaire is designed for children with CP aged 4 to 12 years. There are two versions of the CP QOL-Child questionnaire. The first version contains 53 items and is designed for children aged 9-12 years, which should be filled by children. The second version comprises 66 items specially designed for children aged 4-12 years, which should be filled by parents. The first and second versions contain five and seven domains, respectively. The seven domains are as follows: 1. Acceptance and social wellbeing, 2. Participation and physical health, 3. Functioning, 4. Emotional wellbeing, 5. Pain and feeling about disability, 6. Access to health services, and 7. Family health. The first five domains are common between the two versions. In other words, the last two domains are specific to the second version. The questionnaire was administered through face-to-face interviews with parents. The parents were asked to express their child’s feelings on a 10-point Likert scale ranging from 1 ("not at all happy") to 9 ("very happy") (22). Akbar Fahimi et al. (2012) provided the Persian version of this questionnaire and evaluated its validity and reliability. 

Spearman correlation coefficient between CP QoL and MACS test (r = 0.13-0.40) and between CP QoL and GMFCS (0.18- 0.32) revealed a moderate correlation (23). All surgeries were performed by an orthopedic surgeon team at Akhtar hospital. Then, according to the applied immobilization technique, participants were divided into two equally sized groups of bandage and cast. The sampling process continued until matching the two groups concerning the variables of age and gender. Moreover, it worth noting that all subjects in both groups received a relatively similar conventional rehabilitation program in hospital and *Iran* rehabilitation clinic after surgery.

Postoperative rehabilitation programs included passive, supportive, and active mobilization of organs according to the Bobath method (24). For the cast group, rehabilitation was initiated at 3-4 weeks after surgery, while for the bandage group, it began one day after the surgery. The exercise protocol consisted of seven exercises, including extensors and flexors of the hip, knee, and ankle. For children that could successfully overcome the resistance against gravity, an elastic band was used to increase the resistance (25). In the first and third months after surgery, while the rehabilitation program was continuing, the CP QoL-Child questionnaire was filled again by parents. Data were analyzed using the non-parametric Mann-Whitney test and ANOVA by SPSS version 20.0. 

## Results

Demographic characteristic of participants is presented in Table 1.

**Table1:** Demographic characteristic of participants, separated by the group (n=80)

Immobilization method (Group)	Mean age	Standard deviation	Minimum	Maximum	Number
Bandage	8.85	0.67	8	11	40
Casting	8.85	0.96	7	11	40

Central indicators and dispersion of QoL scores, separated by the study group, following the postoperative immobilization is provided in Table 2.

**Table 2:** The QoL score of pre- and post-operation at the first and third months after the surgery based on the method of immobilization (n=80)

Immobilization method (Group)	Evaluation time	QOL score (%)		Standard deviation	Minimum (%)	Maximum (%)	Number
**Bandaging**	Pre-operation		44.54	5.19	33.6	54.65	40
	Post-operation	First month	45.30	6.24	35.58	57.28	
		Third month	47.14	5.73	35.68	58.6	
**Casting**	Pre-operation		42.30	4.55	32.52	52.54	40
	Post-operation	First month	41.09	4.67	31.52	50.43	
		Third month	44.04	4.36	35.34	52.76	

In the bandage group, the difference in mean QoL score before the surgery and the first month after the surgery was 0.76, which was not statistically significant (p=0.053). For the bandage group, this score was 2.6 three months after surgery. Also, the difference in mean QoL scores at the first month and at the third month after surgery was -1.21 and 1.74, respectively, which was statistically significant p<0.001). Furthermore, there was no significant difference concerning the mean QoL score between the two groups before the surgery (p=0.044). In addition, for both groups, the difference in mean ratio of QoL changes, based on the CP QoL-Child questionnaire, was significant in the first month after the surgery (P<0.00). We found an increase in mean ratio changes of QoL in the bandage group was observed (p<001); but in the cast group, it was -2.86% (chart 1). Furthermore, in the casting group, the postoperative mean ratio change of QoL was decreased significantly (p<0.001). 

**Figure1:**
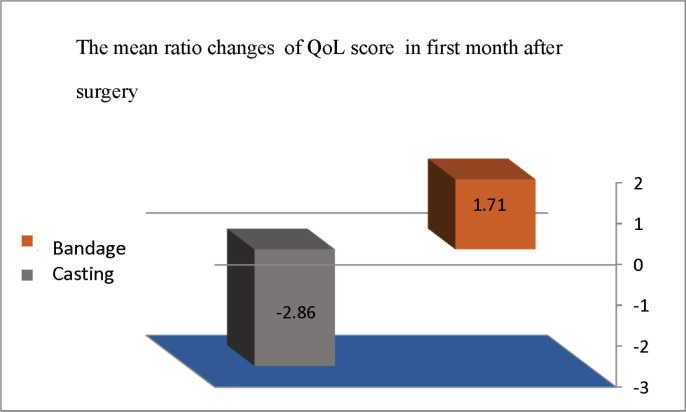
The mean ratio of QoL score changes in the first month after surgery according to the immobilization method

The mean ratio of QoL score changes three months after surgery compared to before surgery is provided in Chart 2. The mean ratio of QoL score changes in the third month after surgery in the bandage and cast groups was increased by 5.83 and 5.06%, respectively. This increase was statistically significant (p<0.001), but the difference in the mean ratio of QoL score changes between the two groups was not significant (p=0.64). 

**Figure2:**
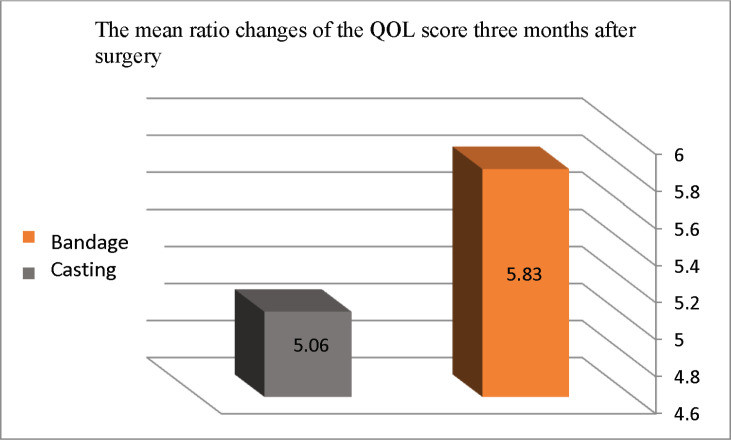
The mean ratio of QoL score changes three months after surgery according to the immobilization method

## Discussion

According to the findings, there was a non-significant increase in the mean ratio of QoL score changes in the bandage group at the first month after surgery. However, in the cast group, the mean ratio of QoL score changes was significantly reduced after surgery. For both methods, the mean ratio of QoL score changes was significantly increased three months after surgery, but the difference between the two groups was not significant. The difference in QoL between the two methods can be attributed to the weight of the bandage, which is lighter than the cast, and also it provides easier transfer and mobility for the child. On the other hand, the bandage can be removed. In addition, not only it can be changed after showering or skin cleaning, but it also promotes personal health and reduces the likelihood of surgical wound infection. Furthermore, the bandage, in contrast to the cast, doesn’t cause problems such as dermal scarring resulting from the pressure of the cast on the skin and the bone pain due to immobilization. As a matter of fact, less pain through applying bandage is associated with higher QoL in children with CP. 

## In Conclusion

In this study, two types of postoperative immobilization techniques (i.e. bandage and casting) were administered. Bandaging is a new method of immobilization, which is not investigated by previous studies. Except for Gupta et al. that, in a study on lower extremity surgery in children with CP, administered compression bandage and superficial skin elasticity techniques to create short stretch after a hip surgery. However, they reported that ten days after surgery, the bandage was removed, and the cast was applied from hip to toe and 4-6 weeks later, the cast was removed, and the rehabilitation program was started (26).

Based on the findings of the present study, both methods of immobilization were associated with improved QoL in children with CP, which is consistent with the studies by Davis et al. (2010), Dickinson et al (2007), Cuomo et al. (2007), and HimPes (2013) (27-30). Gorton et al. compared 75 children with spastic CP, who underwent surgery in order to improve gait, with a non-surgical group. They also evaluated the potential efficacy of surgery outcomes. Twelve months after surgery, the QoL was evaluated using the PedsQL questionnaire. They reported few significant changes in the QoL in the non-surgical group, but for the surgical group, a significant improvement was observed in QoL after one year (31). The minimal change observed in the QoL in the study by Gorton can be attributed to the PedsQL questionnaire, which is not specially designed for CP children. Thomason et al. (2011), in a survey on 19 children with spastic diplagia, have used Child Health Questioner (CHQ) in order to evaluate the QoL and concluded that the QoL did not improve until 12 months after surgery (32). However, in this study, a specific questionnaire (CP QOL-Child) was used to measure the postoperative QoL. According to the results reported by Davis et al. (2010) and Karlon et al (2010), the CP-QoL is specifically designed to focus on perceptional and spiritual aspects of CP children (27-33). 

The minimum time that is considered in previous studies to evaluate the QoL of children has been nine months. However, two time points of one and three months after the surgery were considered in the present study. Himpens et.al didn’t evaluate the impact of time interval after surgery on QoL; they also noted that no previous study had investigated the impact of this variable (29). However, Gage et al. (2009) investigated the effect of time interval after SEMLS surgery on the function of children with CP and reported decreased function as well as an increased dependency in the first month after surgery, but 12 months after the surgery, an improvement was observed in function (34). 

However, this study highlighted significant changes in the mean ratio of the QoL score following administration of both casting and bandaging methods after surgery (p<0.001). 
